# Supplementary material for: Performance of Plasma Phosphorylated tau-217 in Patients on the Continuum of Alzheimer’s Disease
Source: Int J Mol Sci. 2025 Jul 15;26(14):6771. doi: 10.3390/ijms26146771 (PMC12295251; doi:10.3390/ijms26146771)
Supplement: Supplementary file 1 [file ijms-26-06771-s001.zip › ijms-3734329-supplementary.pdf]

Supplementary data

Performance of Plasma phosphorylated tau-217 in patients on the continuum of Alzheimer's disease

Farida Dakterzada[1], Ricard López-Ortega[2], Alba Vilella-Figuerola[1], Nathalia Montero-Castilla[1], Iolanda Riba-Llena[1], Maria Ruiz-Julián[1], Alfonso Arias[1], Jordi Sarto[1], Nuria Tahan[1], Gerard Piñol-Ripoll[1, 3]

(1) Unitat de Trastorns cognitius, Cognition and Behaviour Study Group, Hospital Universitari Santa Maria de Lleida, Universitat de Lleida, IRBLleida, 25198, Lleida, Spain

(2) Laboratori Clinical ICS, Hospital Universitari Arnau de Vilanova, 25198, Lleida, Spain

(3) Unitat d'Alzheimer i Altres Trastorns Cognitius, Hospital Clínic de Barcelona, Fundació de Recerca Clínic Barcelona – Institut d'Investigacions Biomèdiques August Pi i Sunyer (IDIBAPS), 08036, Barcelona, Spain

Suplememntary Table S1. Characteristics of the study population according to the CSF Aβ42/40 status.

	N	All (n =276)	Aβ− (n = 99)	Aβ+ (n = 177)	p-value
**Demographic data**					
Age	276	74 (70;78)	73 (70;78)	75 (71;78)	0.011
Sex	276	164 (59%)	43 (43%)	121 (68%)	< 0.001
Family history of cogniti	274	113 (41%)	40 (40%)	73 (41%)	0.915
Education	264	9 (8;12)	10 (9;14)	12 (8;14)	0.364
Comorbidities					
Hypertension, n(%)	276	153 (55%)	57 (58%)	96 (54%)	0.593
Diabetes, n(%)	276	84 (30%)	28 (28%)	56 (32%)	0.561
Dyslipidemia, n(%)	276	148 (54%)	52 (53%)	96 (54%)	0.784
Depression, n(%)	275	98 (36%)	36 (36%)	62 (35%)	0.815
**Plasma AD biomarkers**					
Aβ40 pg/mL	123	303 (276;359)	312.635 (280.635;396.058)	303.39 (277.72;351.11)	0.935
Aβ42 pg/mL	124	24.03 (21.09;27.84)	26.07 (24.3;32.845)	23.16 (20.23;26.61)	0.003
p-tau181 pg/mL	126	2.3 (1.69;3.13)	1.685 (1.508;2.173)	2.63 (2.17;3.42)	< 0.001
p-tau217 pg/mL	258	0.34 (0.15;0.69)	0.133 (0.11;0.161)	0.436 (0.336;0.76)	< 0.001
Aβ42/40	123	0.08 (0.07;0.08)	0.085 (0.08;0.09)	0.07 (0.07;0.08)	< 0.001
p-tau181/Aβ42	124	0.1 (0.07;0.14)	0.06 (0.06;0.078)	0.11 (0.1;0.15)	< 0.001
p-tau217/Aβ42	108	0.01 (0.01;0.03)	0.005 (0.004;0.006)	0.02 (0.013;0.036)	< 0.001
**CSF AD biomarkers**					
Aβ42 pg/mL	276	546 (411;799)	832 (660;1178)	428 (326;505)	< 0.001
Aβ40 pg/mL	276	10789 (8336;13468)	9985 (8240;13078)	10196 (8056;12246)	0.300
t-tau pg/mL	276	456 (269;748)	213 (158;297)	552 (359;833)	< 0.001
p-tau181 pg/mL	276	69 (42;121)	31 (29;42)	100 (59;132)	< 0.001
Aβ42/40	276	0.05 (0.04;0.08)	0.087 (0.078;0.095)	0.041 (0.036;0.048)	< 0.001
p-tau181/Aβ42	276	0.15 (0.04;0.27)	0.04 (0.03;0.048)	0.23 (0.15;0.32)	< 0.001
t-tau/Aβ42	276	0.9 (0.3;1.58)	0.255 (0.213;0.32)	1.43 (0.89;1.84)	< 0.001
**Other variables**					
BMI	258	26.6 (23.9;29.2)	27.3 (25.2;28.8)	25.4 (23;27.8)	< 0.001
GFR	264	75.4 (62.8;86.3)	70.8 (57.3;84.6)	79.3 (64.5;86.3)	0.854
MMSE	269	25 (21;27)	26 (22;27)	25 (21;27)	< 0.001
APOE4, n(%)	272	106 (38%)	15 (15%)	91 (51%)	< 0.001

Supplementary Table S2. Correlation coefficients between plasma p-tau217 and other plasma and CSF AD biomarkers.

	Biomarker	Correlation coefficient	p-value
Plasma p-tau217	CSF Aβ42	−0.590	< 0.001
	CSF Aβ40	0.022	0.728
	CSF p-tau181	0.728	< 0.001
	CSF t-tau	0.644	< 0.001
	CSF Aβ42/40	−0.707	< 0.001
	CSF p-tau181/Aβ42	0.797	< 0.001
	CSF t-tau/Aβ42	0.76	< 0.001
	Plasma Aβ40	0.101	0.303
	Plasma Aβ42/40	−0.345	< 0.001
	Plasma p-tau181	0.837	< 0.001
	Plasma p-tau181/Aβ42	0.842	< 0.001

Supplementary table S3. Correlation coefficients between plasma p-tau217 and CSF AD biomarkers with the study population stratified by sex..

			CSF biomarkers						
			Aβ42	Aβ40	Aβ42/40	p-tau181	t-tau	p-tau/Aβ42	t-tau/Aβ42
Man	Plasma	r	-0.599	0.006	-0.725	0.641	0.498	0.783	0.725
	p-tau217	p-value	<0.001	0.955	<0.001	<0.001	<0.001	<0.001	<0.001
		N	94	94	94	94	94	94	94
Woman	Plasma	r	-0.494	-0.002	-0.620	0.718	0.665	0.773	0.743
	p-tau217	p-value	<0.001	0.983	<0.001	<0.001	<0.001	<0.001	<0.001
		N	140	140	140	140	140	140	140

Data adjusted for age, APOE4 status, and eGFR

Supplementary Table S4. Correlation coefficients between plasma p-tau217 and CSF AD biomarkers with the study population stratified by APOE4 status

			CSF biomarkers						
			Aβ42	Aβ40	Aβ42/40	p-tau181	t-tau	p-tau/Aβ42	t-tau/Aβ42
APOE4	Plasma	r	-0.587	0.038	-0.731	0.705	0.621	0.789	0.742
non-	p-tau217	p-value	<0.001	0.654	<0.001	<0.001	<0.001	<0.001	<0.001
carrier		N	142	142	142	142	142	142	142
APOE4	Plasma	r	-0.399	-0.049	-0.466	0.563	0.520	0.672	0.657
carrier	p-tau217	p-value	<0.001	0.639	<0.001	<0.001	<0.001	<0.001	<0.001
		N	92	92	92	92	92	92	92

Data adjusted for age, sex, and eGFR

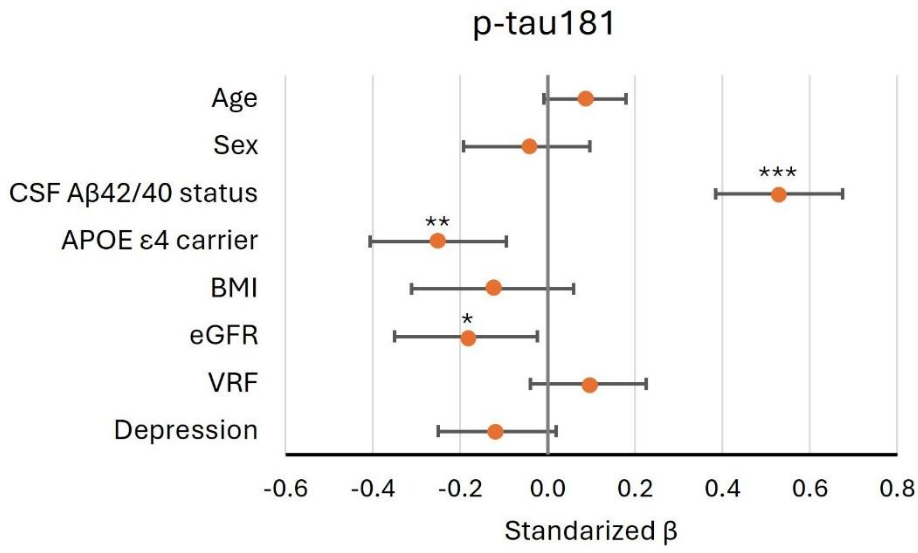

Supplementary Figure S1. Association between plasma levels of p-tau181 and potential confounding variables. Significant associations were observed between plasma levels of p-tau181 and CSF Ab42/40 status (β = 0.530, 95% CI 0.385–0.674, p < 0.001), harboring APOE

e4 allele ($\beta = -0.250$, 95% CI: − 0.406 to −0.095, $p = 0.002$), and eGFR (($\beta = -0.187$, 95% CI: −0.350 to −0.023, $p = 0.026$).
